# The Hydrogen Bonded Structures of Two 5-Bromobarbituric Acids and Analysis of Unequal C5–X and C5–X′ Bond Lengths (X = X′ = F, Cl, Br or Me) in 5,5-Disubstituted Barbituric Acids

**DOI:** 10.3390/cryst6040047

**Published:** 2016-04-22

**Authors:** Thomas Gelbrich, Doris E. Braun, Stefan Oberparleiter, Herwig Schottenberger, Ulrich J. Griesser

**Affiliations:** 1 Institute of Pharmacy, University of Innsbruck, Innrain 52c, 6020 Innsbruck, Austria; 2 Institute of General, Inorganic and Theoretical Chemistry, University of Innsbruck, Innrain 80, 6020 Innsbruck, Austria

**Keywords:** crystal structure, hydrogen bond, topology, geometry optimization, barbiturates

## Abstract

The crystal structure of the methanol hemisolvate of 5,5-dibromobarbituric acid (**1MH**) displays an H-bonded layer structure which is based on N–H⋯O=C, N–H⋯O(MeOH) and (MeOH)O–H⋯O interactions. The barbiturate molecules form an H-bonded substructure which has the **fes** topology. 5,5′-Methanediylbis(5-bromobarbituric acid) **2**, obtained from a solution of 5,5-dibromobarbituric acid in nitromethane, displays a N–H⋯O=C bonded framework of the **sxd** type. The conformation of the pyridmidine ring and the lengths of the ring substituent bonds C5–X and C5–X′ in crystal forms of 5,5-dibromobarbituric acid and three closely related analogues (X = X′ = Br, Cl, F, Me) have been investigated. In each case, a conformation close to a C5-endo envelope is correlated with a significant lengthening of the axial C5–X′ in comparison to the equatorial C5–X bond. Isolated molecule geometry optimizations at different levels of theory confirm that the C5-endo envelope is the global conformational energy minimum of 5,5-dihalogenbarbituric acids. The relative lengthening of the axial bond is therefore interpreted as an inherent feature of the preferred envelope conformation of the pyrimidine ring, which minimizes repulsive interactions between the axial substituent and pyrimidine ring atoms.

## 1 Introduction

**Scheme 1:**
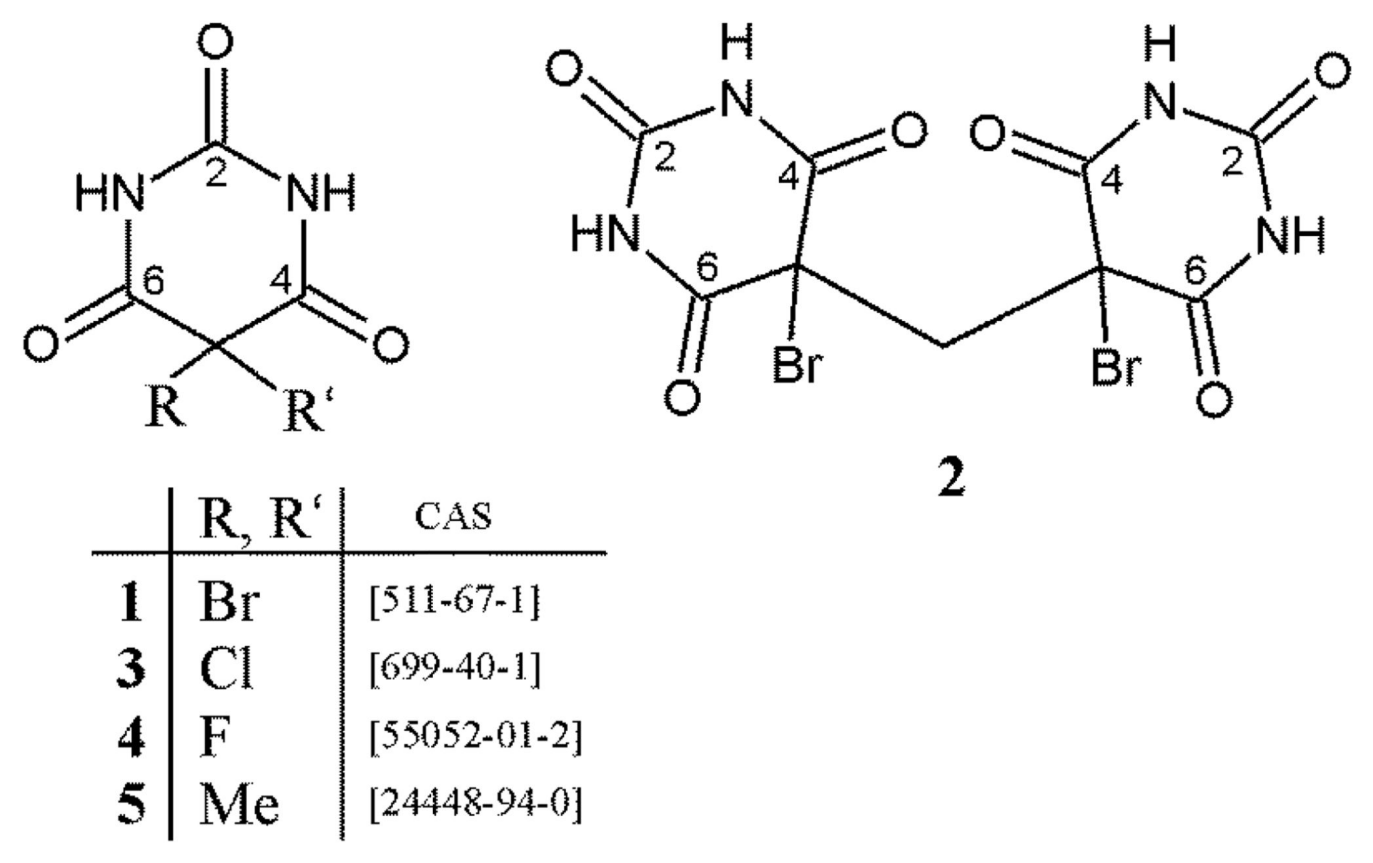
Structure formulas of compounds discussed in this report.

Barbiturates are derivatives of barbituric acid which have the ability to act as nervous system depressants. A number of 5,5-disubstituted species have been used widely as sedative, hypnotic and anticonvulsant agents [1–4]. These barbiturates are known for their high propensity to crystallize in multiple solid forms, and they are a model polymorphic system in which a set of competing H-bonded structures (HBSs) occurs. In the course of our systematic study of this group of compounds [5–9], we have investigated polymorphs of 5,5-dibromobarbituric acid (**1**) and 5,5-dichlorobarbituric acid (**3**), some of which had been first described by Groth more than 100 years ago [10]. The polymorphs of **1** and **3** display four distinct 1D, 2D or 3D hydrogen-bond motifs, and three of these motifs are unique in that they have not been encountered in any of the more than 50 known crystal structures of analogous barbiturates bearing two organic groups as ring substituents R and R′ at atom C5 (Scheme 1) [11,12]. Few crystal structures of solvates and hydrates of 5,5-disubstituted barbiturates have been reported so far [7,13–16]. The formation of H-bond motifs which are distinct from those of the corresponding unsolvated species is expected in cases where additional H-bond donor and/or acceptor functions are present in the solvent molecule.

Herein we report the crystal structure of the methanol hemisolvate of dibromobarbituric acid (**1MH**) and that of a new derivative, 5,5′-methanediylbis(5-bromobarbituric acid) (**2**) (Scheme 1), obtained from an unexpected reaction of **1** with nitromethane. The complex HBSs of these crystals will be discussed in detail and a specific feature of the 5,5-dibromobarbituric acid molecule in the crystal structure of **1MH**, namely the unequal lengths of the two C5–Br bonds, will be analyzed by means of comparison with crystal structures of structural analogues and their geometry optimized molecular structures. Additionally, a low-temperature redetermination of the crystal structure of 5,5-dimethylbarbituric acid (**5**) is reported.

## 2 Results and Discussion

### 2.1 Crystal Structure of 5,5-Dibromobarbituric Acid Hemisolvate (*1MH*)

**Figure 1:**
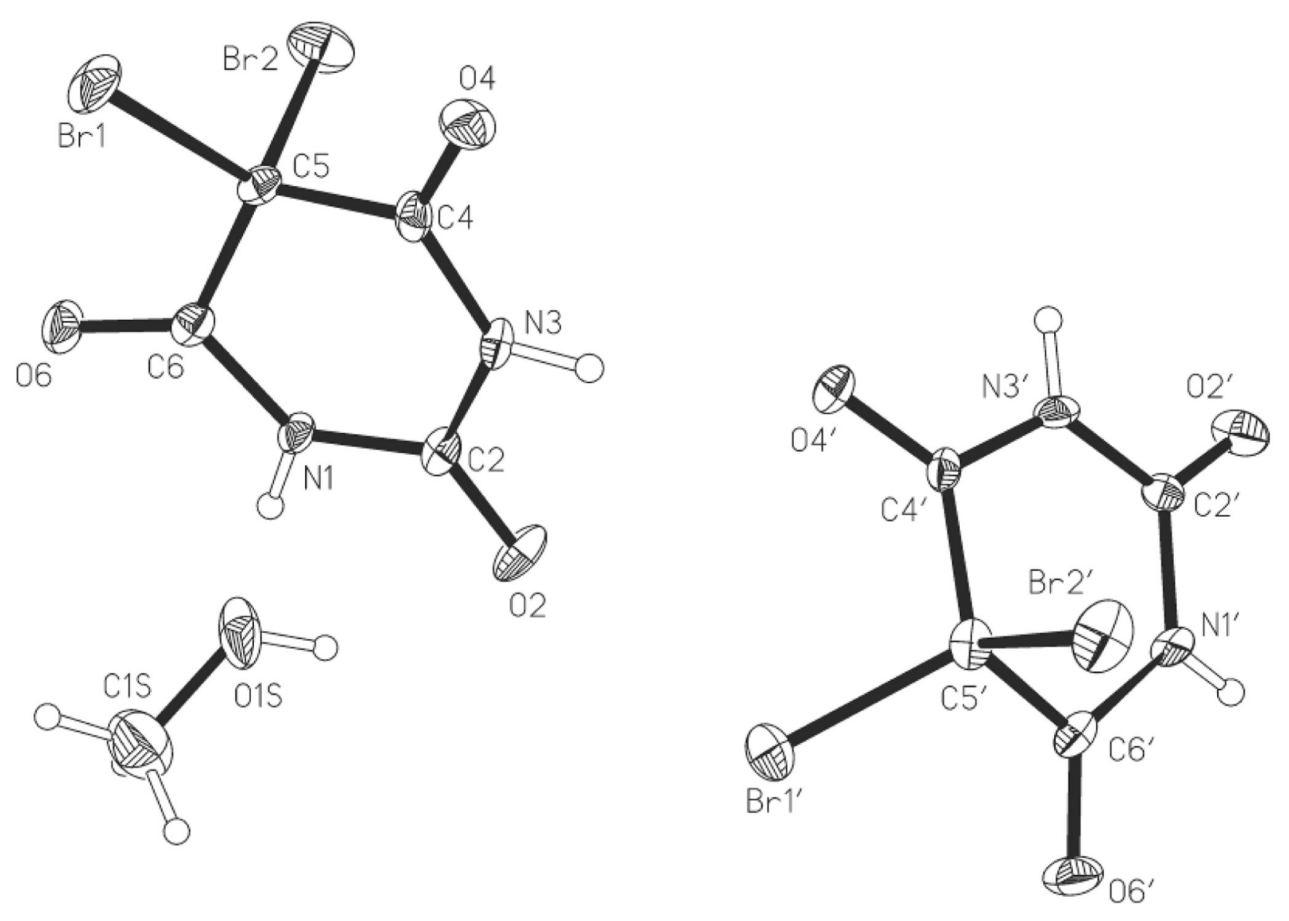
Asymmetric unit of **1MH**. Thermal ellipsoids are drawn at the 50% probability level. H-atoms are drawn as spheres of arbitrary size.

The title structure has the monoclinic space group symmetry *P*2_1_/*n* (Table 1). Its asymmetric unit (Figure 1) consists of two dibromobarbituric acid molecules (labeled A and B) and one MeOH molecule. The pyrimidine rings adopt a C5-endo envelope conformation, characterized by these ring puckering parameters according to Cremer and Pople [17]: *Q* = 0.201(4) Å, θ = 50.7(11)°, ϕ = 242.9(14)° (molecule A) and *Q* = 0.196(4) Å, θ = 139.9(12)°, θ = 61.6(17)° (molecule B). The positions of the atoms C5 and C5′ of A and B, respectively, deviate by 0.437(4) and 0.450(4) Å from the mean plane defined by the other five atoms of the pyrimidine ring. In both molecules, the bond distance to the equatorial bromo substituent, C5–Br1, 1.907(4) Å, and C5′–Br1′, 1.912(4) Å, is significantly shorter than that to the axial substituent, C5–Br2, 1.963(4) Å, and C5′–Br2′, 1.967(3) Å.

**Figure 2:**
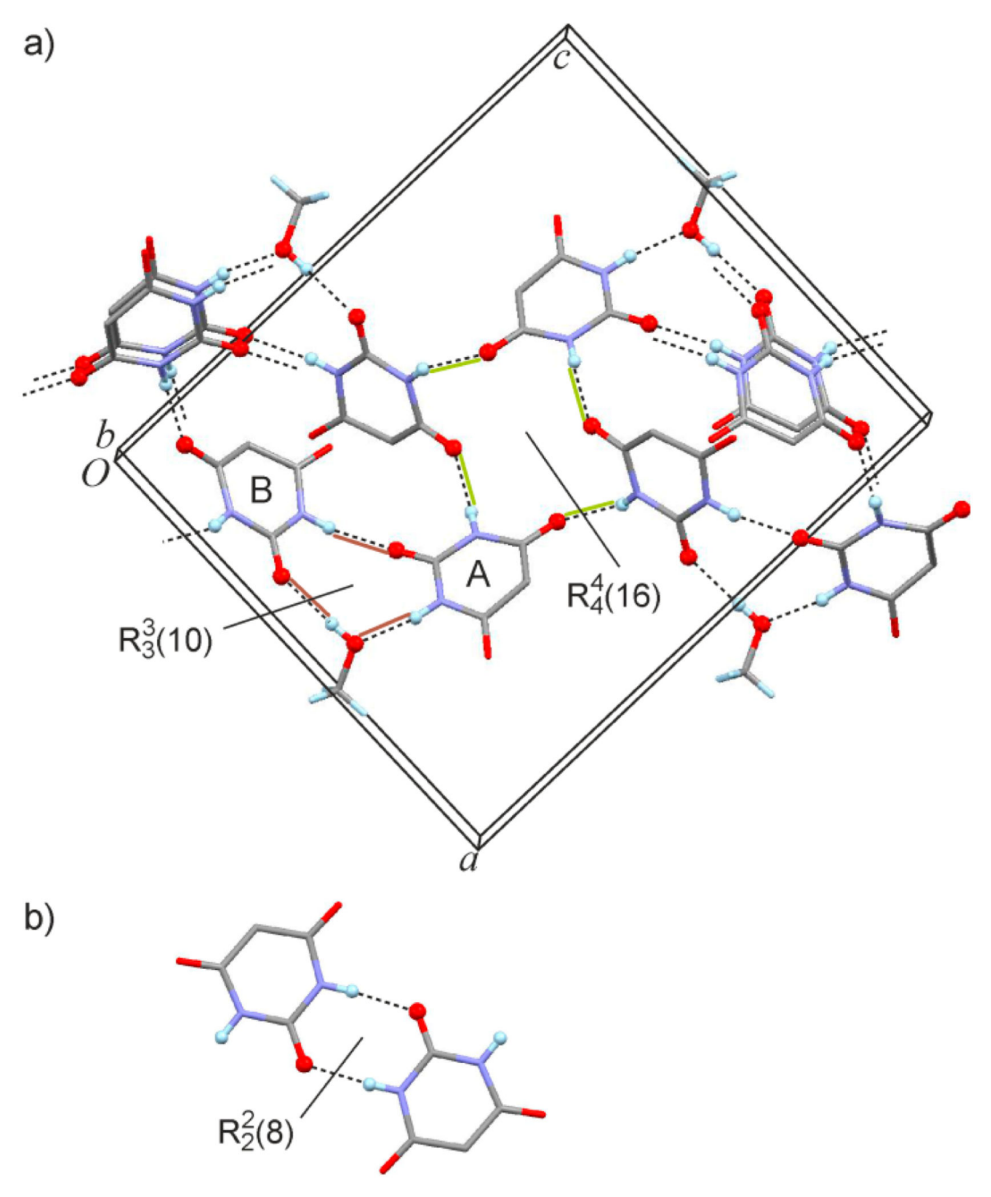
(**a**) N–H⋯O=C, N–H⋯O(MeOH) and (MeOH)O–H⋯O=C-bonded layer structure of **1MH**. O and H atoms involved in hydrogen bonding are drawn as spheres and hydrogen bonds are drawn as dashed lines. Br atoms are omitted for clarity; (**b**) ${\text{R}}_{2}^{2}$(8) ring motif involving the C2 carbonyl functions of two barbiturate molecules, found in the orthorhombic and monoclinic polymorphs of **1**.

Molecules A and B are each H-bonded, via one-point connections, to four other molecules (Table 2). However, the two molecule types are distinct with respect to their H-bond connectivity. Via its NH H-bond donor groups, molecule A is linked to one B molecule, N3–H⋯O=C4′, and to one MeOH molecule, N1–H⋯O=C(MeOH). Additionally, it is connected via its C2 and C4 carbonyl functions, to another two B-type molecules. By contrast, both NH groups of a B-type molecule link to A-molecules, N1′–H⋯O=C2^i^ and N3′–H⋯O=C4^ii^. Moreover, its C4′ carbonyl group links to a third A-molecule and the C2′ group to a MeOH molecule. The latter serves as a bridge between an A and a B molecule in such a way that ${\text{R}}_{3}^{3}(10)$ rings [18,19] are generated (Figure 2a). The resulting HBS is a layer structure which lies parallel to $\left(10\bar{1}\right)$ and also contains ${\text{R}}_{4}^{4}(16)$ rings which involve two A and two B molecules. The latter ring motif connecting four barbiturate molecules is not encountered in either the orthorhombic or the monoclinic polymorph of **1**, both of which display rings connecting six H-bonded molecules via N–H⋯O=C interactions. However, the ${\text{R}}_{3}^{3}(10)$ ring is reminiscent of the ${\text{R}}_{2}^{2}(8)$ motif via the C2 carbonyl function (Figure 2b), which is present in both previously described polymorphs of **1** (the orthorhombic polymorph **1a** is the desolvation product of **1MH**, see Figure S1 of the Supplementary Materials).

**Figure 3:**
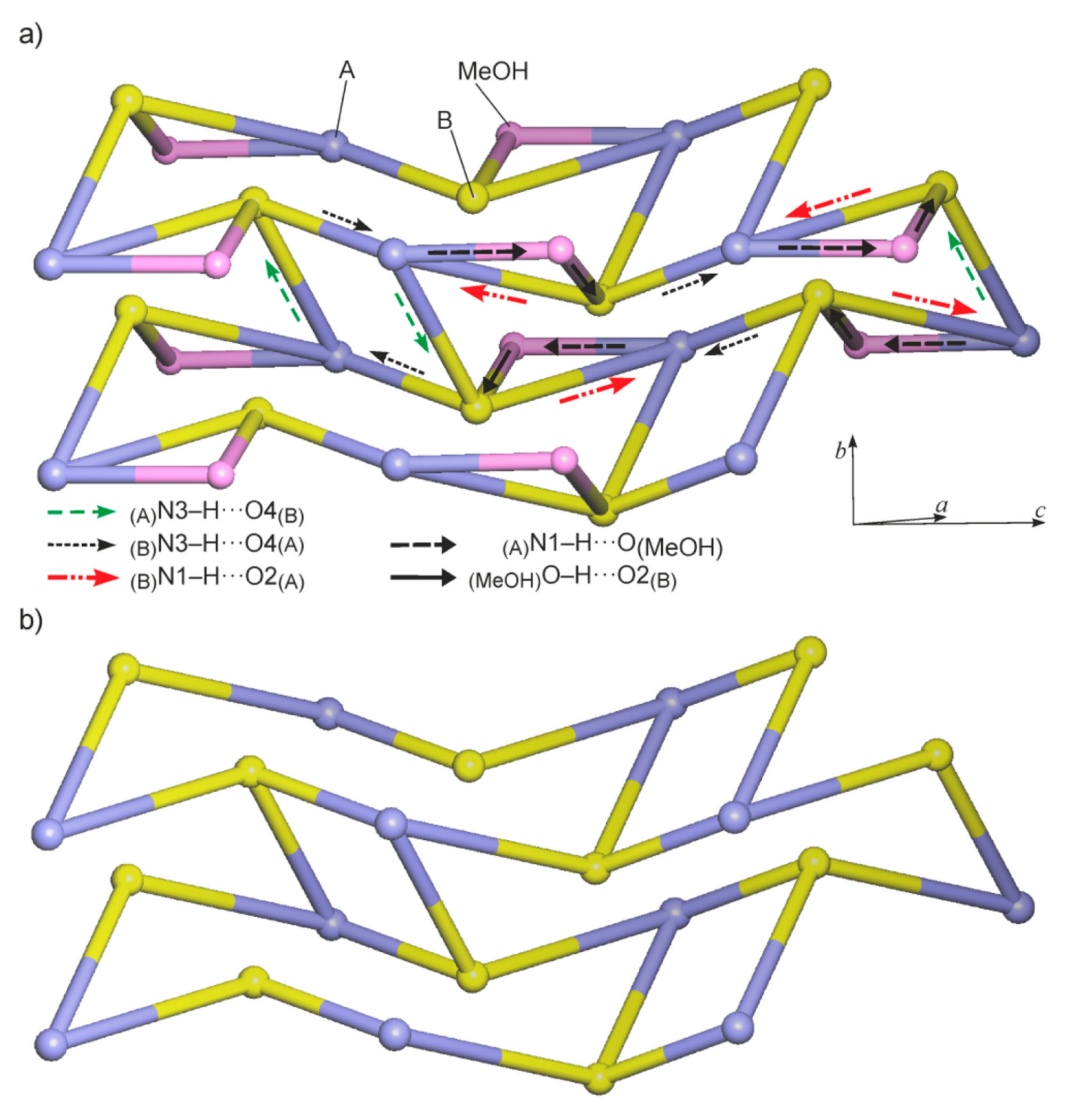
HBS of **1MH**, repretented as a graph according to ref. [21]: (**a**) the complete L(4_4_)_2_.2_2_[(3.4.8^2^.9^2^)_2_(3)] structure and (**b**) the L(4_4_)_2_[4.8^2^-**fes**] substructure formed by the H-bonded molecules of **1**.

The diagram in Figure 3 is based on the topological net of the HBS of **1MH**, generated in the manner described by Baburin & Blatov [20]. The nodes represent molecules, and links between these nodes represent H-bond connections. As proposed by Hursthouse *et al.* [21], arrows have been added to indicate the type and direction of each H-bond interaction. Only this latter modification reveals the subtle differences between the two molecule types A and B (Figure 3a, Table 2) with respect to their H-bond connectivity. If the bridging MeOH molecules are not taken into account, the barbiturate molecules alone form a 2-periodic 3-connected uninodal subnet which has the 4.8^2^-**fes** topology [22] (Figure 3b). The topology symbol for the complete H-bonded net composed of 4-connected A and B molecules and 2-connected MeOH (Figure 3a) is (3.4.8^2^.9^2^)_2_(3). The short symbol according to Hursthouse *et al*. [21] for the complete HBS is L(4_4_)_2_.2_2_[(3.4.8^2^.9^2^)_2_(3)], and for the A + B substructure it is L(4_4_)_2_[4.8^2^-**fes**]. The stacking of multiple H-bonded layers in the crystal is shown in Figure S2 of the Supplementary Materials.

### 2.2 Crystal Structure of 5,5′-Methanediylbis(5-bromobarbituric acid)(2)

**Figure 4:**
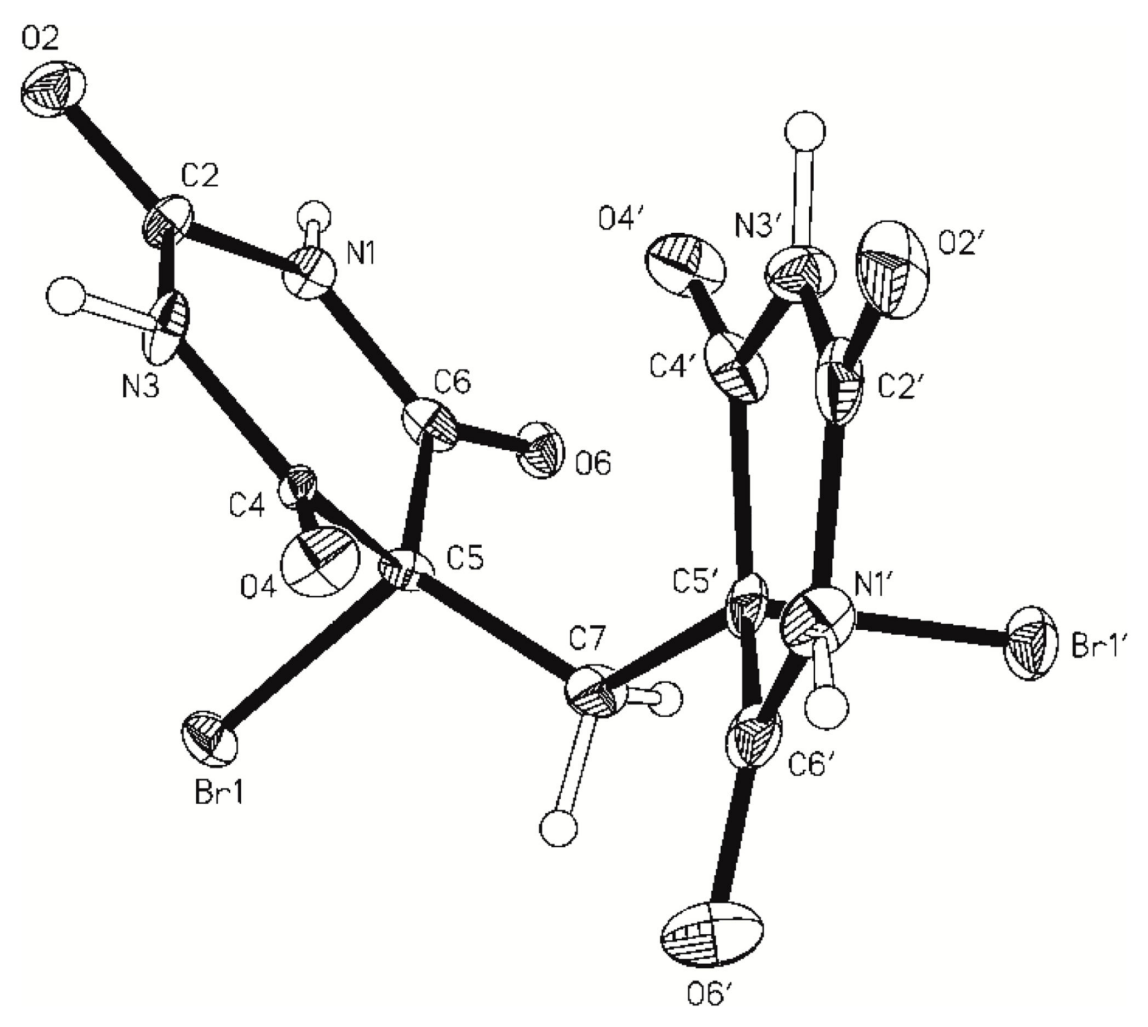
Molecular structure of **2**. Thermal ellipsoids are drawn at the 70% probability level and H-atoms drawn as spheres of arbitrary size.

This compound has the orthorhombic space group symmetry *P*2_1_2_1_2_1_ (Table 1), and its asymmetric unit consists of a single molecule (Figure 4). The two six-membered rings adopt C5-endo envelope conformations of the same handedness. The Cremer-Pople puckering parameters are Q = 0.240(9) Å, θ = 65(2)°, ϕ = 224(2)° for the pyrimidinetrione unit A (N1, C2, N3, C4, C5, C6), and they are Q = 0.156(10) Å, θ = 45(2)°, ϕ = 235(5)° for unit B (N1′, C2′, N3′, C4′, C5′, C6′). The axial C5–Br1 and C5′–Br1′ bond distances of 1.973(8) Å and 1.982(8) are in good agreement with the corresponding axial values found in **1MH**. The angle between the mean planes of the two heterocyclic rings is 50.1(3)°.

**Figure 5:**
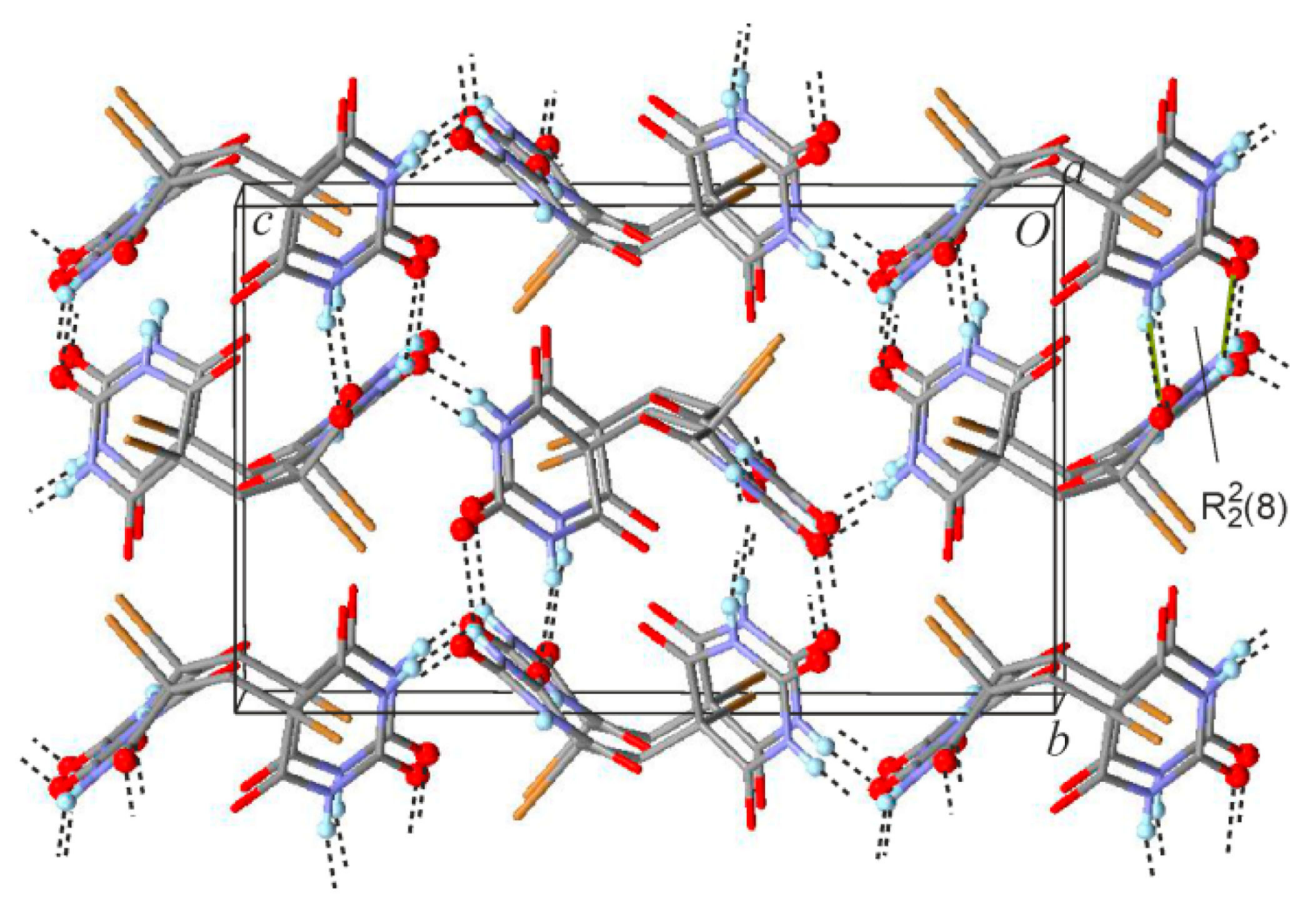
N–H⋯O=C-bonded framework of **2**. O and H atoms involved in hydrogen bonding are drawn as spheres and hydrogen bonds are drawn as dashed lines.

**Figure 6:**
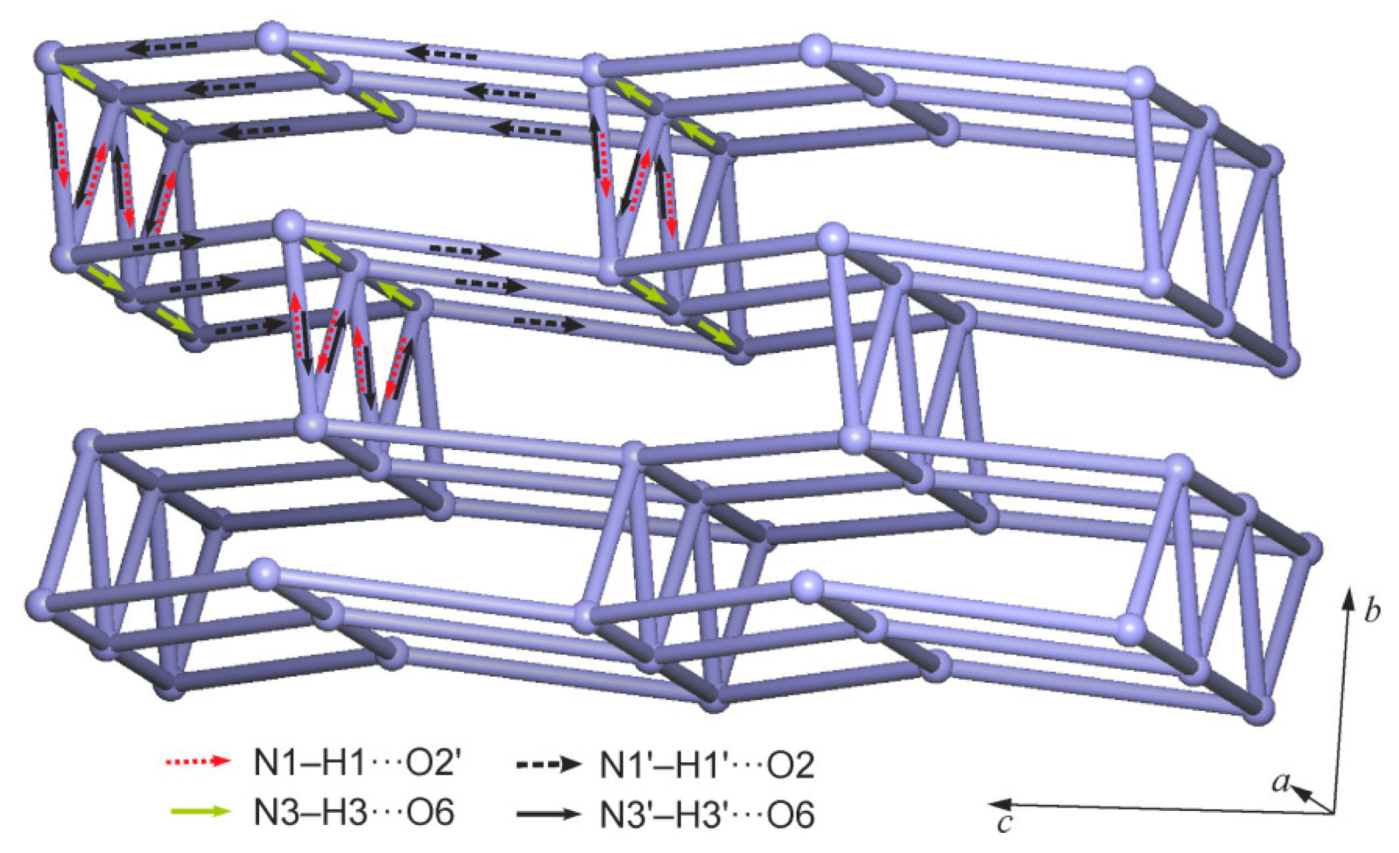
Graph according to ref. [21] for the HBS of **2** with the symbol F6_4_[3^3^.4^6^.5^5^.6**-sxd**].

Each molecule is linked to six neighboring molecules via four one-point and two two-point N–H⋯O=C connections. The two independent pyrimidinetrione units differ somewhat from one another in their H-bond connectivity (Table 3). Two-point connections between neighboring molecules involve the C6 and C2 carbonyl groups of the A and B rings, N1–H1⋯O2′^i^ and N3′–H3′⋯O6^iv^, resulting in an asymmetrical ${\text{R}}_{2}^{2}(8)$ ring. With the second H-bond donor function, rings A and B are connected to the C6 and C2 carbonyl groups, respectively, of A-type rings belonging to two different molecules, N3–H3⋯O6^ii^ and N1′–H1′⋯O2^iii^. As a result, the C2 carbonyl functions of both rings and the C6 carbonyl group of ring A are engaged in one and two H-bonds, respectively, whereas the C6 carbonyl group of ring B is not involved in N–H⋯O=C bonding. The HBS resulting from all these interactions is an H-bonded framework (Figure 5) which has the topology of the 3^3^.4^6^.5^5^.6**-sxd** net [22]. Figure 6 shows a graphical representation of this HBS, whose short symbol according to ref. [21] is F6_4_[3^3^.4^6^.5^5^.6**-sxd**]. As in the structure of **1MH**, each NH group is engaged in exactly one N–H⋯O=C interaction, but the resulting H-bonded framework is completely different from the H-bonded layer structure of **1MH**.

The crystal of **2** contains two intermolecular contacts Br1⋯O2(1 − *x*, *y* + 0.5, 1.5 − *z*) and Br1′⋯O2′(*x* − 1, *y*, *z*) whose Br⋯O distances of 2.81 and 2.98 Å, respectively, are considerably smaller than the sum of the van der Waals radii of Br and O (3.37 Å [23]), and the corresponding C–Br⋯O angles are 165° and 174° (Supplementary Materials, Figure S3). We have used the semi-classical density sums (SCDS-PIXEL) [24–27] method to assess the importance of these contacts. This method enables to calculate total energy contributions (*E*_T_) associated with individual molecule–molecule pairs, which are partitioned into contributions from Coulombic (*E*_C_), polarization (*E*_P_), dispersion (*E*_D_) and repulsion (*E*_R_) effects. Table S1 of the Supplementary Materials shows the results of these calculations for the seven most important pairs of symmetry-equivalent molecule–molecule interactions. The most stabilizing interaction pair (denoted as #1/1′ in Table S1) accounts for more than 33% of the total PIXEL energy of the crystal, *E*_T,Cry_, of −152.3 kJ·mol^−1^ and involves two-point N–H⋯O-bond connections between neighboring molecules (based on the interactions #a and #d in Table 3). This is followed by two pairs of symmetry-equivalent molecule–molecule interactions involving one-poini N–H⋯O-bond connections (#b and #c in Table 3) which contribute 19% (#3/3′) and 18% (#5/5′) to *E*_T,Cry_. Two pairs of molecule–molecule interactions (#7/7′ and #9/9′), which are dominated by dispersion effects due to extensive van der Waals contacts, each account for 13% of *E*_T,Cry_. By contrast, the stabilization effect of a single molecule–molecule interaction involving the aforementioned short Br1⋯O2(1 − *x*, *y* + 0.5, 1.5 − *z*) contact is very small (*E*_T_ = −1.5 kJ·mol^−1^) as its significant Coulombic energy of −16.9 KJ·mol^−1^ is counterbalanced by strong repulsion (31.8 kJ·mol^−1^). As a result, the corresponding symmetry-equivalent interaction pair (#13/13′) affects *E*_T,Cry_ by just 1%. Effects arising from the second short Br⋯O contact, Br1′⋯O2′(*x* − 1, *y*, *z*) in (#3′/3), are overlaid by those of the N3–H3⋯O6(*x*, *y* + 1, *z*) bond. However, a comparison with the other interaction pair (#5/5′) involving a one-point N–H⋯O-bond connection indicates that any additional stabilization due to the short Br⋯O contact should be very small. Overall, the SCDS-PIXEL calculations indicate that the two short Br⋯O contacts contribute only little to the stabilization of the lattice in **2**.

### 2.3 Analysis of C5–X and C5–X′ Bond Lengths (X = X′ = F, Cl, Br, Me) in 5,5-Disubstituted Barbituric Acids

As reported above, the 5,5-dibromobarbituric acid molecule in **1MH** shows a significant disparity between its two C5–Br bond distances as well as a C5-endo envelope conformation. The elongated axial bond could be the result of the specific spatial characteristics of the axial substituent position. As such, it could be interpreted as an effort to avoid repulsive close interactions between the electron clouds of the axial substituent and pyrimidine ring atoms, specifically C4 and C6 (the corresponding Br ∙ ∙ ∙ C distances for the axial and equatorial Br atom are 2.77–2.79 Å and 2.83–2.85 Å, respectively). In order to test this hypothesis we will first establish whether a similar correlation between equatorial and axial C5–X and C5–X′ bond distances on the one hand and pyrimidine-ring puckering parameters on the other hand can be found for the previously reported [11] polymorphic forms **1a** and **1b**. The same kind of analysis will also be carried out for the crystal structures of the dichloro, difluoro and dimethyl analogues of **1** contained in the Cambridge Structural Database (CSD; version 5.37 [28]). Additional theoretical calculations of molecular structures in the gas phase were carried out in order to ascertain the possible influence of crystal packing effects (see next section).

Table 4 contains information on the pucker of pyrimidine rings (derived from Cremer-Pople parameters [17]) and the corresponding equatorial (*d*_C5–X_) and axial (*d*_C5–X′_) bond distances for the polymorphs of dibromo- (**1a, 1b** [11]) and dichlorobarbituric (**3a, 3b** [11], **3c** [12]) acid and for the crystal structures of difluoro- and dimethylbarbituric acid (**4** [29], **5** [30]). As the difference between *d*_C5–X_ and *d*_C5–X′_ in the room-temperature structure of **5** is relatively small a redetermination at 173 K (Table 1) was carried out in an effort to obtain very accurate data.

All pyrimidine rings listed in Table 4 adopt either an envelope conformation with C5 at the flap or a conformation which is very near to it. The puckering amplitudes *Q* lie between 0.14 and 0.26 Å. The equatorial C5–X bond is always significantly shorter (taking into account standard uncertainties) than the axial C5–X′ bond. The difference Δ*d* between axial and equatorial distances are 0.027–0.066 Å for (X, X′ = Br), 0.020 – 0.050 Å for (X, X′ = Cl), 0.35 Å (for X, X′ = F) and 0.041 Å for (X, X′ = Me). A survey of Br–C(sp^3^)–Br structure fragments contained in the CSD [28] indicates that the systematic differences between C5–Br and C5–Br′ bonds distances found in **1MH**, **1a** and **1b** are unusual (see Figure S4 of the Supplementary Materials).

### 2.4 Geometry Optimization of 5,5-Disubstituted Barbituric Acid Analogues (X = X′ = F, Cl, Br, Me)

The molecular structures of the four 5,5-disubstituted barbituric acid analogues (X = X′ = Br, Cl, F, Me) were optimized in gas phase and the calculated parameters (Table 5) have been contrasted to the observed conformations. The C5-endo envelope was calculated to be the conformational energy minimum for all three halogen-substituted barbituric acid analogues, independent of the used level of theory. In each case, it is correlated with a lengthening of the axial relative to the equatorial bond. These results indicate that the non-planarity of the pyrimidine ring is not an effect of crystal packing, *i.e.* of intermolecular forces. Instead, it is associated with the energetically preferred molecular structure. The computed puckering amplitudes *Q* and the difference between axial and equatorial distances are in agreement with the experimental values for the halogen barbituric acid analogues listed in Table 4. Except for X = X′ = Me, changing the basis set from 6-31G(d,p) to aug-cc-pVTz results in slightly smaller puckering amplitudes (Table 5).

The MP2/6-31G(d,p) optimized molecular structure of 5,5-dimethylbarbituric acid is also a C5-endo envelope conformation, as observed in the experimental crystal structure, and is in agreement with previous calculations performed at the MP2/6-31G(d) level of theory [30]. However, using a DFT (PBE0) method with different basis sets, a nearly planar pyrimidine ring and Δ*d* values of less than 0.01 Å were obtained. In a previous report, Roux and coworkers have shown that the planar conformation becomes more stable than the C5-endo envelope if another DFT method, B3LYP, and a different basis set or the combination MP2/6-31(3df,2p) are used [30]. The disagreement between the methods indicates that the steric effect of the Me group on the ring conformation is less pronounced than that of a halogen substituent. Moreover, a conformational change of the 5,5-dimethylbarbituric acid molecular structure from planar to C5-endo envelope geometry and vice versa requires less energy than the same change in each of the halogen analogues.

### 2.5 Correlation between Bond Parameters

**Figure 7:**
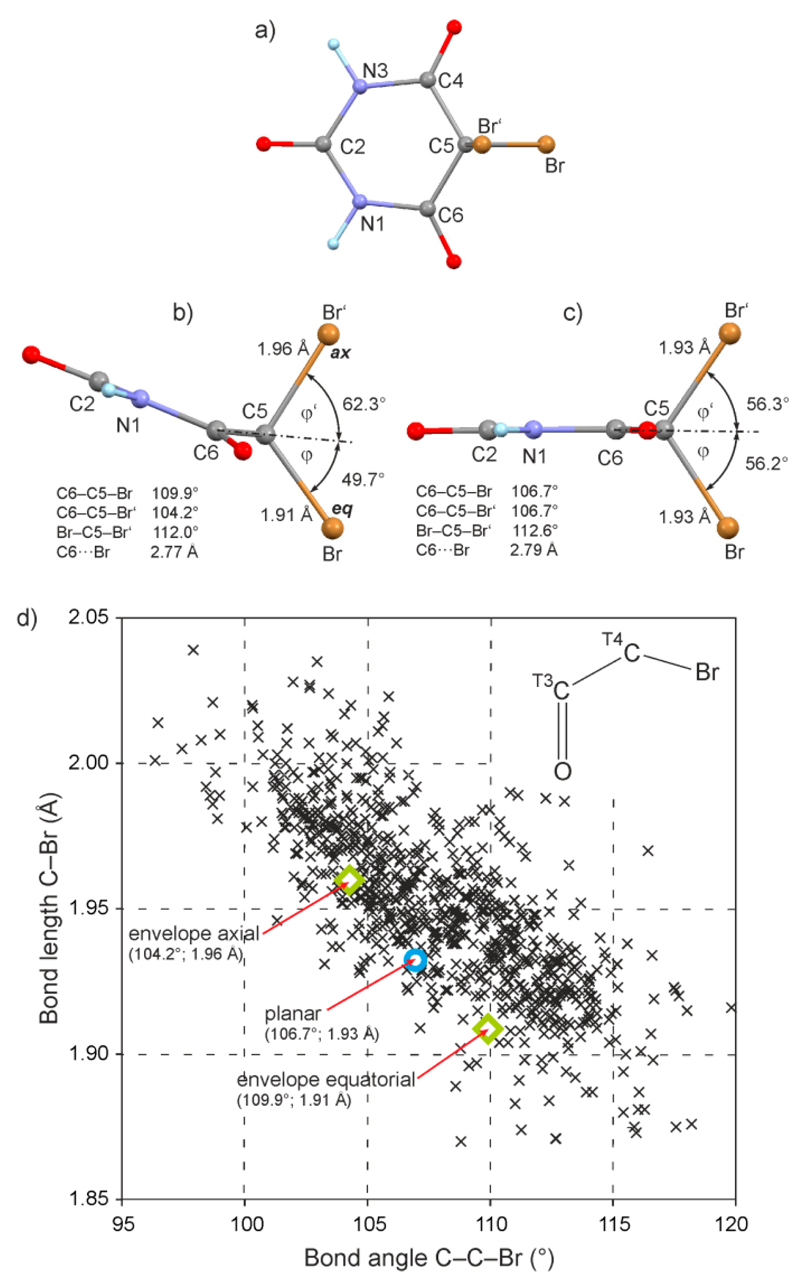
(**a**) Numbering scheme for **1** (gray = C, light blue = H, blue = N, red = O, brown = Br). (**b**) PBE0/aug-cc-pVTz optimized molecular structure of **1** showing a C5-endo envelope conformation of the pyrimidine ring [view parallel to the plane defined by (N1, C2, N3, C4, C6), with N3 and C4 superimposed by N1 and C6, respectively; the trace of the plane defined by (C4, C5, C6) is drawn as a dash-dot line; φ and φ′ are defined as the angles formed between this plane and the equatorial C5–Br and the axial C5–Br′ bond, respectively]; (**c**) analogous view of a PBE0/aug-cc-pVTz optimized molecular structure of **1** with constrained planar ring geometry. (**d**) The C–Br bond lengths in 819 O=C(sp^2^)–C(sp^3^)–Br fragments (inset in upper right-hand corner; from 541 crystal structures with *R* < 0.075, no disorder, no errors, not polymorphic, no ions, no powder structures, only organics) plotted against the corresponding C–C–Br bond angle. The bond parameters of the molecular structures shown in (**b**) and (**c**) are represented by the green rhombuses and the blue circle, respectively.

The optimized molecular structure of **1**, which has the C5-endo envelope conformation, is depicted in Figure 7b. A corresponding molecular structure of **1**, obtained from an alternative optimization with a constrained planar ring geometry, is shown in Figure 7c. In the latter structure, the Br–C5–Br′ bond angle is bisected by the trace of the plane defined by ring atoms C4, C5 and C6 so that all four bond angles of the type C4/C6–C5–Br/Br′ are 106.7°. The bond geometry around C5 changes significantly in the C5-envelope conformation in that the plane defined by C5, Br and Br′ is rotated by more than 6° against the plane defined by C5, C4, and C6 so that the axial C5–Br′ bond moves towards the axis of the ring. As the orientation of the C5–Br and C5–Br′ bonds relative to one another remains almost unchanged, the two axial C4/6–C5–Br′ bond angles are decreased by 2.5°. The ensuing shortening effect on the axial 1,3-distances C∙ ∙ ∙ Br′ is largely counterbalanced by the simultaneous elongation of the C5–Br′ bond by 0.03 Å (envelope: C∙ ∙ ∙ Br′ = 2.77 Å; planar: C∙ ∙ ∙ Br′ = 2.79 Å). This suggests that the relative lengthening of the axial C5–Br′ bond helps to prevent unfavorably close 1,3-contacts between the electron clouds of the axial substituent and the ring atoms C4 and C6. This relative lengthening would therefore be a steric effect of the energetically preferred C5-endo envelope conformation. Moreover, larger equatorial C4/6–C5–Br angles are accompanied by a shortening of the equatorial bond C5–Br by 0.02 Å in comparison to the molecule with a planar pyrimidine ring.

In order to establish the general correlation between the C–Br bond length and the corresponding Br–C–C bond angle (or the 1,3-distance C∙ ∙ ∙ Br), we have carried out a survey of the CSD [28] in which crystal structures containing the O=C(sp^2^)–C(sp^3^)–Br fragment have been considered. The C–Br bond lengths of 819 such structure fragments are plotted against the corresponding Br–C–C bond angles in Figure 7d. The distribution of data points in the relevant interval between 110° and 100° indicates that a decrease in the Br–C–C angle is generally correlated with a lengthening of the C–Br bond. All data points for the two optimized molecules of **1** shown in Figure 7b,c agree very well with this general trend.

**Figure 8:**
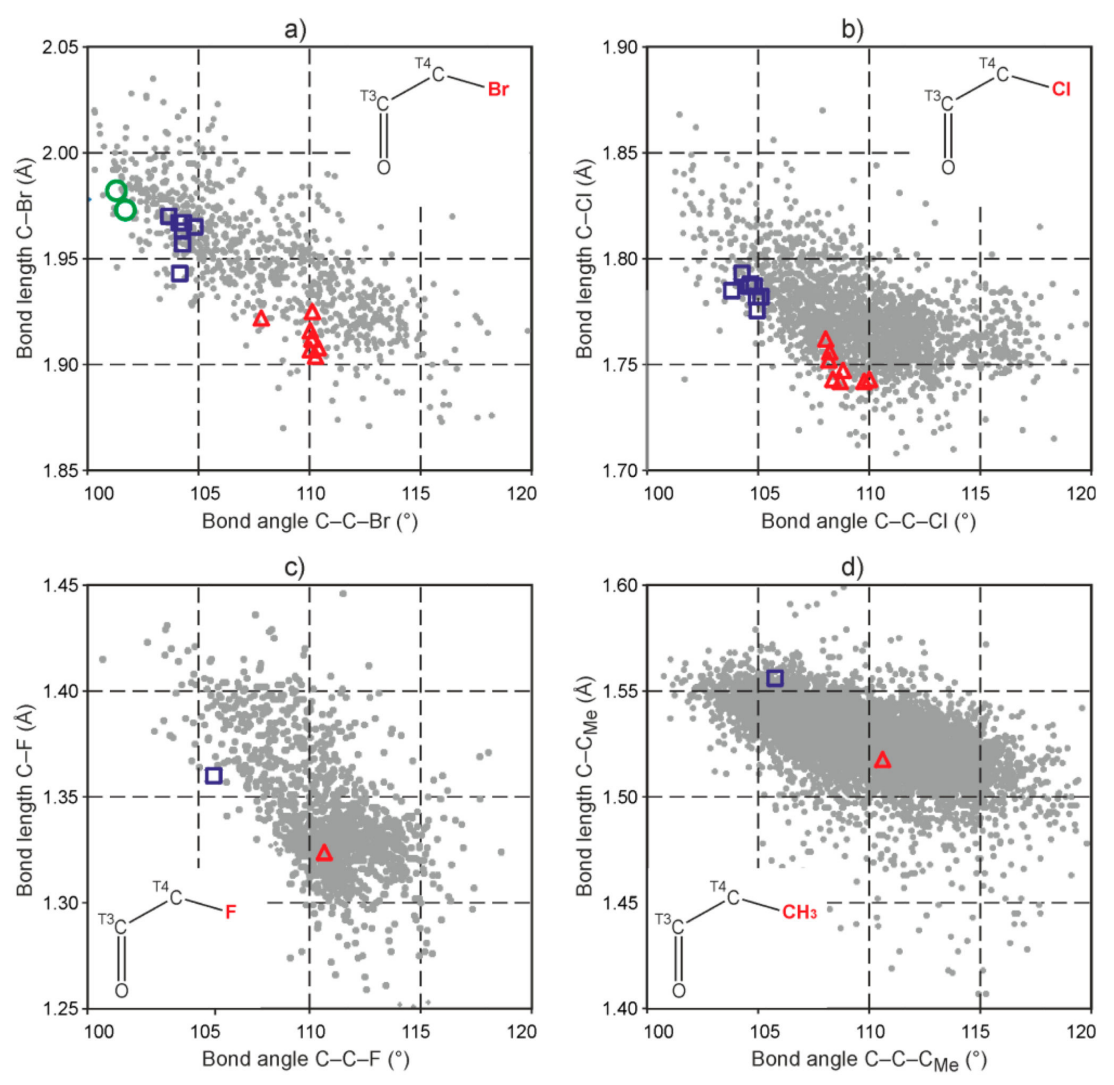
Bond lengths C–X plotted against bond angles C–C–X (gray dots) for structure fragments O=C(sp^2^)–C(sp^3^)–X with X = Br (**a**); Cl (**b**); F (**c**) and CH_3_ (**d**) identified in CSD surveys. Red triangles and blue squares represent the parameters of the equatorial C5–X and axial C5–X′ bonds, respectively, listed in Table 4. Green circles in (**a**) represent data points for the axial C5–Br ring substituent bonds of the two C5-endo envelope rings in the molecule of **2**.

Similar surveys have also been carried out for the analogous O=C(sp^2^)–C(sp^3^)–X fragments with X = Cl, F, Me. The resulting distance *vs.* angle plots (Supplementary Materials, Figure S5) show that smaller C–C–X bond angles are correlated with longer C–X bonds in the 110° to 100° range in each case. The diagrams in Figure 8 were obtained by superimposing these plots with the experimental data for barbiturates listed in Table 4. In each case, the bond parameters of the equatorial as well as axial ring substituents agree well with the general trend. This is also true for the axial C5–Br bonds of each of the two C5-endo envelope rings in the molecule of **2** (Figure 8a).

## 3 Experimental Section

### 3.1 Preparation of Crystal Forms

5,5-Dibromobarbituric acid (**1**) was purchased from Sigma-Aldrich, St. Louis, MO, USA (European affiliate, Steinheim, Germany). Crystals of **1MH** were obtained, at room temperature, from a solution of **1** in MeOH. Desolvation of **1MH** on air resulted in the orthorhombic polymorph **1a** (Supporting Information, Figure S1).

Single crystals of 5,5′-methanediylbis(5-bromobarbituric acid) (**2**) were obtained as products of an unexpected reaction of **1** in a nitromethane solution upon storage at room temperature for several weeks. An NMR tube with a perforated cap was used as a crystallization vessel in order to achieve a slow evaporation rate. The newly introduced methylene linker obviously originates from the nitromethane solvent. Considering the highly acidic character of nitromethane (including its tautomeric equilibrium between *aci*-form and *nitro*-form), it seems likely that, as a first step, a 5-bromo-5-nitromethylbarbituric acid intermediate was formed by nucleophilic replacement of one Br atom by a nitromethane anion. Regardless of the specific mode of dimerization and the nature of the organic nitroalkane reactant involved, the final methylene moiety of **2** would have to be cleaved from the nitro functionality. Such a conversion, e.g., with nitrite as a leaving group, would represent a kind of retro-Kornblum reaction that has been documented in numerous reports [31]. However, 5,5-disubstituted barbituric acid derivatives have also been described to exhibit an extraordinary rich chemistry, and in particular the first bromo group is known to be extremely active [32,33]. Therefore, the direct monodebromination of **1** by a nitromethane tautomer to form 5-bromobarbituric acid may also be considered the source of a potential participant in the formation of the dibromo dimer (Supplementary Materials, Scheme S1).

In summary, the nitromethane anion may be capable of bromobarbiturate alkylation or may serve as a debrominative reducing agent. In the absence of specific investigations, any mechanistic proposal remains however highly speculative. It is therefore unsurprising that our attempts to reproduce compound **2** according to any of the anticipated dimerization steps have been unsuccessful or inconclusive. Nevertheless, an independent synthetic route has been successfully established which starts from methylene bridged barbituric acid (CAS [27406-39-9]) [34] and involves direct bromination in glacial acetic acid of the preformed, already bridged system (Supplementary Materials, Scheme S2). The identity of the resulting precipitate with the investigated single crystal phase of **2** formed in nitromethane was confirmed by comparison of its X-ray powder pattern with the powder pattern calculated from the single crystal data of **2**. A full NMR-spectroscopic characterization of **2** was not possible due to its low solubility. The heating of **2** in solvents suitable for NMR measurements (caried out to achieve better solubility) resulted in a conversion to spiro[furo[2,3-*d*]pyrimidine-6(2*H*),5′(2′*H*)-pyrimidine]-2,2′ (CAS [1333529-88-6]) [35,36]. The identity of the elimination product was unequivocally confirmed by a single-crystal structure determination, which will be published in due course.

5,5-Dimethylbarbituric acid (**5**) was purchased from Sigma-Aldrich, St. Louis, MO, USA (European affiliate, Steinheim, Germany). Single crystals of **5** were obtained by sublimation on a hot bench at 230 °C.

### 3.2 Single-Crystal X-ray Structure Analyses

Intensity data were collected, using Mo radiation (λ = 0.71073 Å), on an Oxford Diffraction Gemini-R Ultra diffractometer operated by the CrysAlis software [37]. The data were corrected for absorption effects by means of comparison of equivalent reflections using the program *SADABS* [38]. The structures were solved using the direct methods procedure in *SHELXS97* [39] and refined by full-matrix least squares on *F*^2^ using *SHELXL-2014* [40]. Non-hydrogen atoms were refined anisotropically. Hydrogen atoms were located in difference maps. All NH hydrogen atoms were refined with distance restraints of N–H = 0.86(2) Å and hydrogen atoms bonded to C atoms were refined using riding models. In the case of **1MH,** the O–H distance in the MeOH moiety was restrained to 0.86(2) Å, the *U*_iso_ parameters of NH hydrogen atoms were refined freely and those of H atoms in the solvent molecule were set to 1.2*U*_eq_ (OH group) or 1.5*U*_eq_ (CH_3_ group) of the parent atom. In the structure of **2**, the *U*_iso_ parameters of all hydrogen atoms were set to 1.2*U*_eq_ of the parent N or C atom. The *U*_iso_ parameters of all hydrogen atoms in the crystal structure of **5** were refined freely.

CCDC 1441623–14416235 contains the supplementary crystallographic data for this paper. These data can be obtained free of charge via http://www.ccdc.cam.ac.uk/conts/retrieving.html (or from the CCDC, 12 Union Road, Cambridge CB2 1EZ, UK; Fax: +44 1223 336033; E-mail: deposit@ccdc.cam.ac.uk)

### 3.3 Analysis of Crystal Data

Puckering parameters for pyrimidine rings were calculated with *PLATON* [41]. The topology of hydrogen-bonded structures was determined and classified with the programs *ADS* and *IsoTest* of the *TOPOS* package [42] in the manner described by Baburin & Blatov [20].

### 3.4 Computational Modelling

Gas phase *ab initio* geometry optimizations for each of the four 5,5-dibsubstited barbituric acids (X = X′ = Br, Cl, F, Me) were performed at the PBE0/6-31G(d,p), PBE0/aug-cc-pVTz and MP2/6-31G(d,p) levels of theory using GAUSSIAN09 [43].

### 3.5 SCDS-PIXEL Calculation

Intermolecular interaction energies for **2** were calculated with the SCDS-PIXEL [24–27] method and the program *OPiX* [44]. The structure model of the CIF was used, and C–H and N–H distances were re-calculated to standard lengths within *OPiX.* No optimization of the molecular geometry was performed. An electron density map was calculated on a three-dimensional grid with a step size of 0.08 Å at the MP2/6-31G(d,p) level using GAUSSIAN09 [43]. A PIXEL condensation factor of 4 was applied, giving superpixels with dimensions 0.32 × 0.32 × 0.32 Å. The calculations yielded interaction energies partitioned into Coulombic, polarization, dispersion and repulsion terms with an expected accuracy of 1 – 2 kJ · mol^−1^.

## 4 Conclusions

The complex H-bonded structure of **1MH** is derived from the 4.8^2^-**fes** net which is a well-known topology of two-dimensional MOFs [45], while the HBS of **2** is based on the 3^3^.4^6^.5^5^.6-**sxd** framework, which has been previously identified as a frequent topology type in organic crystals [20]. The ${\text{R}}_{3}^{3}\left(10\right)$ rings in the H-bonded structure of **1MH** are reminiscent of the ${\text{R}}_{2}^{2}\left(8\right)$ motif which has been found in the crystal structures of two polymorphs of 5,5-dibromobarbituric acid (**1a, 1b)** and in those of many other barbiturates. It seems therefore possible that the desolvation of **1MH** and subsequent formation of the orthorhombic polymorph **1a** proceeds via a direct conversion of ${\text{R}}_{3}^{3}\left(10\right)$ into ${\text{R}}_{2}^{2}\left(8\right)$ rings. The minimum energy molecular conformation of 5,5-dihalogen substituted derivatives of barbituric acid is the C5-endo envelope geometry in which the two axial angles C4/C6–C5–X′ are significantly smaller than the corresponding equatorial C4/C6–C5–X angles. Simultaneously, the relatively long axial C5–X′ bond (in comparison with the equatorial C5–X bond) prevents unfavorably short 1,3-distances (C4⋯X′ and C6⋯X′) between the axial substituent and pyrimidine ring atoms. This interpretation of the axial C5–X′ and equatorial C5–X bond distances is consistent with general trends in the correlation between the C–C–X bond angles and C–X bond lengths of O=C(sp^2^)–C(sp^3^)–X structure fragments (X = Br, Cl, F, Me) contained in the CSD.

## Supplementary Materials

The following are available online at http://www.mdpi.com/2073-4352/6/4/47/s1, a comparison of the PXRD characteristics of the desolvation product of **1MH** with those of polymorph **1a**, an additional diagram of the H-bonded structure of 1MH, details of the SCDS-PIXEL calculation for **2**, results of CSD surveys and information about the synthetic procedure for compound **2**.

Supplementary material

## Figures and Tables

**Table 1:** Crystallographic details for **1MH**, **2** and **5**.

Compound	1MH	2	5
Moiety formula	2(C_4_H_2_Br_2_N_2_O_3_)· CH_4_O	C_9_H_6_Br_2_N_4_O_6_	C_6_H_8_N_2_O_3_
Formula mass	603.83	426.00	156.14
Crystal system	monoclinic	orthorhombic	triclinic
Space group	*P*2_1_/*n*	*P*2_1_2_1_2_1_	$p\bar{1}$
*Z*	4	4	2
*a*/Å	14.6451(4)	6.8347(3)	5.6667(7)
*b*/Å	6.73660(16)	10.6206(5)	6.4172(7)
*c*/Å	17.1159(4)	17.0982(9)	10.5800(8)
α/°	90	90	84.429(8)
β/°	90.238(2)	90	81.341(8)
γ/°	90	90	64.916(12)
Unit cell volume/Å^3^	1688.61(7)	1241.13(10)	344.24(7)
Temperature/K	173	173	173
No. of reflections measured	5387	9500	2223
No. of independent reflections	3388	2256	1329
*R_int_*	0.0277	0.1130	0.0268
No. of parameters	238	202	117
Absolute structure parameter (Flack)	-	0.014(18)	-
Final *R*_1_ value (*I* > 2*σ*(*I*))	0.0310	0.0477	0.0331
Final *wR*(*F*^2^) value (all data)	0.0620	0.1220	0.0821

**Table 2:** Geometric parameters for hydrogen bonds in **1MH**.

Type	*D*–H⋯*A*	*d*(*D*–H)/Å	*d*(H⋯*A*)/Å	*d*(*D*⋯*A*)/Å	∠(*D*H*A*)/°
A→MeOH	N1–H1⋯O1S	0.855(10)	1.881(15)	2.721(4)	167(5)
A→B	N3–H3⋯O4′	0.862(10)	2.071(15)	2.910(4)	164(3)
B→A	N1′–H1′⋯O2 i	0.851(10)	1.974(13)	2.814(4)	169(4)
B→A	N3′–H3′⋯O4 ii	0.850(10)	2.162(19)	2.942(4)	152(3)
MeOH→B	O1S–H1S⋯O2′ iii	0.838(10)	1.97(2)	2.757(4)	157(5)

Symmetry transformations: ^i^ –*x* + 1/2, *y* + 1/2, –*z* + 1/2; ^ii^ –*x* + 1, –*y* + 1, –*z* + 1; ^iii^ –*x* + 1/2, *y* – 1/2, –*z* + 1/2.

**Table 3:** Geometric parameters for hydrogen bonds in **2**.

#	Type	*D*–H⋯*A*	*d*(*D*–H)/Å	*d*(H⋯*A*)/Å	*d*(*D*⋯*A*)/Å	*∠*(DHA)/°
a	A→B	N1–H1⋯O2′ i	0.863(14)	2.34(7)	3.085(11)	144(10)
b	A→A	N3–H3⋯O6 ii	0.861(14)	2.13(5)	2.929(10)	153(10)
c	B→A	N1′–H1′⋯02 iii	0.861(14)	2.12(5)	2.933(11)	158(10)
d	B→A	N3′–H3′⋯O6 iv	0.859(14)	2.05(4)	2.871(10)	161(11)

Symmetry transformations: ^i^ *x* – 1/2, –*y* + 1/2, –*z* + 2; ^ii^ *x* + 1, *y*, *z*; ^iii^ –*x* + 3/2, –*y* + 1, *z* + 1/2; ^iv^ *x* + 1/2, –*y* + 1/2, –*z* + 2.

**Table 4:** Conformation and puckering amplitudes *Q* [17] of pyrimidine rings, equatorial (*d*_C5-X_) and axial (*d*_C5-X′_) bond distances at ring atom C5 for the independent molecules (A, B …) in the crystal structures of dibromobarbituric acid **(1)** and several close analogues (3–5; see Scheme 1).

Structure	CSD Refcode	Reference	X, X′	Mol.	Ring Conformation a	Q (Å)	*d*_C5-X_ (Å)	*d*_C5-X′_ (Å)	Δ*d* (Å) b
**1MH**	-	*this work*	Br	A	E	0.201(4)	1.907(4)	1.963(4)	0.056(5)
				B	E	0.196(4)	1.912(4)	1.967(3)	0.055(5)
**1a**	UXIZAD	[11]	Br	A	E	0.217(9)	1.916(8)	1.943(8)	0.027(11)
				B	E	0.091(9)	1.922(8)	1.957(8)	0.035(11)
				C	E→HC	0.232(9)	1.925(9)	1.967(9)	0.042(12)
**1b**	UXIZAD01	[11]	Br	A	E→C	0.184(6)	1.904(6)	1.970(5)	0.066(8)
				B	E→SB	0.246(6)	1.908(5)	1.965(5)	0.057(7)
**3a**	UXIYOQ	[11]	Cl	A	E→SB	0.146(4)	1.762(3)	1.782(3)	0.020(5)
				B	E→SB	0.154(4)	1.743(3)	1.787(3)	0.044(5)
				C	E→SB	0.143(4)	1.756(3)	1.782(3)	0.026(4)
				D	E→SB	0.156(4)	1.742(3)	1.788(3)	0.046(5)
**3b**	UXIYOQ01	[11]	Cl	A	E→HC	0.220(2)	1.742(2)	1.7849(19)	0.043(3)
				B	E	0.112(2)	1.7521(18)	1.7755(18)	0.023(3)
				C	E→HC	0.256(2)	1.743(2)	1.793(2)	0.050(3)
**3c**	UXIYOQ02	[12]	Cl	-	SB→E	0.136(3)	1.7472(18)	1.7868(18)	0.040(3)
**4**	HEKTIA	[29]	F	-	E	0.173	1.324(6)	1.359(7)	0.035(9)
**5** (173 K)	-	*this work*	Me	-	E	0.201(2)	1.518(2) c	1.5586(18) c	0.041(3)
**5** (293 K)	NUXTAC	[30]	Me	-	E	0.200	1.532(3) c	1.562(3) c	0.030(4)

a E = C5-endo envelope; HC = half chair; C = chair; SB = skew boat.

b Δ*d* = *d*_C5-X′_ − *d*_C5-X_.

c Length of the C5-C or C5-C′ bond.

**Table 5:** Calculated parameters, conformation and puckering amplitudes *Q* [17] of pyrimidine rings and the corresponding equatorial (*d*_C5–X_) and axial (*d*_C5–X′_) bond distances at ring atom C5, derived from gas phase *ab initio* models for four 5,5-disubstituted barbituric acid analogues (X = X′ = F, Cl, Br, Me).

Compound (X, X′)	Level of Theory	Ring Conformation a	*Q* (Å)	*d*_C5–X_ (Å)	*d*_C5–X′_ (Å)	Δ*d* (Å) b
**1** (Br)	PBE0/6-31G(d,p)	E	0.252	1.902	1.967	0.064
	PBE0/aug-cc-pVTz	E	0.174	1.909	1.959	0.050
	MP2/6-31G(d,p)	E	0.288	1.919	1.976	0.057
**3** (Cl)	PBE0/6-31G(d,p)	E	0.245	1.748	1.800	0.052
	PBE0/aug-cc-pVTz	E	0.203	1.744	1.794	0.050
	MP2/6-31G(d,p)	E	0.274	1.745	1.797	0.051
**4** (F)	PBE0/6-31G(d,p)	E	0.251	1.331	1.363	0.032
	PBE0/aug-cc-pVTz	E	0.210	1.327	1.360	0.033
	MP2/6-31G(d,p)	E	0.233	1.344	1.378	0.033
**5** (Me)	PBE0/6-31G(d,p)	nearly planar	-	1.535	1.541	0.006
	PBE0/aug-cc-pVTz	nearly planar	-	1.531	1.539	0.008
	MP2/6-31G(d,p)	E	0.197	1.522	1.543	0.021

a E = C5-endo envelope.

b Δ*d* = *d*_C5–*X*′_ — *d*_C5–*X*_.

## References

[1] Brandstätter-Kuhnert M, Aepkers M (1962). Molecular compounds, crystalline solid solutions, and new cases of polymorphism in barbiturates. I. Microchim Acta.

[2] Brandstätter-Kuhnert M, Aepkers M (1962). Molecular compounds, crystalline solid solutions, and new cases of polymorphism in barbiturates. II. Microchim Acta.

[3] Brandstätter-Kuhnert M, Aepkers M (1963). Molecular compounds, crystalline solid solutions, and new cases of polymorphism in barbiturates. III. Microchim Acta.

[4] Kuhnert-Brandstätter M, Vlachopoulos A (1967). Molecular compounds, crystalline solid solutions, and new cases of polymorphism in barbiturates. IV. Microchim Acta.

[5] Zencirci N, Gelbrich T, Kahlenberg V, Griesser UJ (2009). Crystallization of metastable polymorphs of phenobarbital by isomorphic seeding. Cryst Growth Des.

[6] Rossi D, Gelbrich T, Kahlenberg V, Griesser UJ (2012). Supramolecular constructs and thermodynamic stability of four polymorphs and a co-crystal of pentobarbital (nembutal). CrystEngComm.

[7] Zencirci N, Griesser UJ, Gelbrich T, Kahlenberg V, Jetti RKR, Apperley DC, Harris RK (2014). New solvates of an old drug compound (phenobarbital): Structure and stability. J Phys Chem B.

[8] Gelbrich T, Meischberger I, Griesser UJ (2015). Two polymorphs of 5-cyclohexyl-5-ethylbarbituric acid and their packing relationships with other barbiturates. Acta Crystallogr Sect C-Struct Chem.

[9] Gelbrich T, Braun DE, Griesser UJ (2016). Specific energy contributions from competing hydrogen-bonded structures in six polymorphs of phenobarbital. Chem Cent J.

[10] Groth P (1910). Chemische Krystallographie Dritter Teil Aliphatische und Hydroaromatische Kohlenstoffverbindungen.

[11] Gelbrich T, Rossi D, Häfele CA, Griesser UJ (2011). Barbiturates with hydrogen-bonded layer and framework structures. CrystEngComm.

[12] Gelbrich T, Rossi D, Griesser UJ (2012). Tetragonal polymorph of 5,5-dichlorobarbituric acid. Acta Crystallogr Sect E-Struct Rep Online.

[13] Williams PP (1973). Polymorphism of phenobarbitone: The crystal structure of 5-ethyl-5-phenylbarbituric acid monohydrate. Acta Crystallogr Sect B-Struct Sci.

[14] Bhatt PM, Desiraju GR (2007). 5,5-Dibenzylbarbituric acid monohydrate. Acta Crystallogr Sect E-Struct Rep Online.

[15] Gelbrich T, Rossi D, Griesser UJ (2010). Butallylonal 1,4-dioxane hemisolvate. Acta Crystallogr Sect E-Struct Rep Online.

[16] Ravi Kiran B, Suchetan PA, Amar H, Vijayakumar GR (2015). Crystal structure of 5,5-bis(4-methylbenzyl) pyrimidine-2,4,6(1*H*,3*H*,5*H*)-trione monohydrate. Acta Crystallogr Sect E-Struct Commun.

[17] Cremer D, Pople JA (1975). General definition of ring puckering coordinates. J Am Chem Soc.

[18] Etter MC, MacDonald JC, Bernstein J (1990). Graph-set analysis of hydrogen-bond patterns in organic crystals. Acta Crystallogr Sect B-Struct Sci.

[19] Bernstein J, Davis RE, Shimoni L, Chang N-L (1995). Patterns in hydrogen bonding: Functionality and graph set analysis in crystals. Angew Chem Int Ed.

[20] Baburin IA, Blatov VA (2007). Three-dimensional hydrogen-bonded frameworks in organic crystals: A topological study. Acta Crystallogr Sect B-Struct Sci.

[21] Hursthouse MB, Hughes DS, Gelbrich T, Threlfall TL (2015). Describing hydrogen-bonded structures; topology graphs, nodal symbols and connectivity tables, exemplified by five polymorphs of each of sulfathiazole and sulfapyridine. Chem Cent J.

[22] O'Keeffe M, Peskov MA, Ramsden SJ, Yaghi OM (2008). The reticular chemistry structure resource (RCSR) database of, and symbols for, crystal nets. Acc Chem Res.

[23] Bondi A (1964). Van der waals volumes and radii. J Phys Chem.

[24] Dunitz JD, Gavezzotti A (2005). Molecular recognition in organic crystals: Directed intermolecular bonds or nonlocalized bonding?. Angew Chem Int Ed.

[25] Gavezzotti A (2007). Molecular Aggregation: Structure Analysis and Molecular Simulation of Crystals and Liquids.

[26] Gavezzotti A (2005). Calculation of lattice energies of organic crystals: The PIXEL integration method in comparison with more traditional methods. Z Kristallogr.

[27] Gavezzotti A (2005). Quantitative ranking of crystal packing modes by systematic calculations on potential energies and vibrational amplitudes of molecular dimers. J Chem Theory Comput.

[28] Groom CR, Allen FH (2014). The Cambridge Structural Database in retrospect and prospect. Angew Chem Int Ed.

[29] DesMarteau DD, Pennington WT, Resnati G (1994). Fluorinated barbituric acid derivatives. Acta Crystallogr Sect C-Cryst Struct Commun.

[30] Roux MV, Notario R, Foces-Foces C, Temprado M, Ros F, Emel'yanenko VN, Verevkin SP (2010). Experimental and computational thermochemical study and solid-phase structure of 5,5-dimethylbarbituric acid. J Phys Chem A.

[31] Kornblum N, Smiley RA, Blackwood RK, Iffland DC (1955). The mechanism of the reaction of silver nitrite with alkyl halides. The contrasting reactions of silver and alkali metal salts with alkyl halides. The alkylation of ambident anions. J Am Chem Soc.

[32] Brown DJ, Mason SF (2009). The Chemistry of Heterocyclic Compounds: The Pyrimidines.

[33] Grundke G, Keese W, Rimpler M (1985). 5,5-Dibrombarbitursäure, ein neues Reagenz zur Bromierung von gesättigten und α,β-ungesättigten Carbonylverbindungen. Chem Berichte.

[34] Gysling H, Schwarzenbach G (1949). Metallindikatoren II. Beziehungen zwischen Struktur und Komplexbildungsvermögen bei Verwandten des Murexids. Helv Chim Acta.

[35] Jalilzadeh M, Pesyan NN, Rezaee F, Rastgar S, Hosseini Y, Sahin E (2011). New one-pot synthesis of spiro[furo[2,3-d]pyrimidine-6,5′-pyrimidine]pentaones and their sulfur analogues. Mol Divers.

[36] Ara T, Khan KZ (2014). Synthesis of some derivatives of dimedone, γ-pyrone and barbituric acid. J Pharm Res (Mohali, India).

[37] Oxford Diffraction Ltd (2003). Crysalis CCD and CrysAlis RED.

[38] Sheldrick GM (2007). SADABS. Version 2007/7.

[39] Sheldrick GM (2008). A short history of SHELX. Acta Crystallogr Sect A-Fundam Crystallogr.

[40] Sheldrick G (2015). Crystal structure refinement with SHELXL. Acta Crystallogr Sect C-Cryst Struct Chem.

[41] Spek A (2009). Structure validation in chemical crystallography. Acta Crystallogr Sect D-Biol Crystallogr.

[42] Blatov VA (2006). Multipurpose crystallochemical analysis with the program package TOPOS. IUCr Compcomm Newsl.

[43] Frisch MJ, Trucks GW, Schlegel HB, Scuseria GE, Robb MA, Cheeseman JR, Scalmani G, Barone V, Mennucci B, Petersson GA (2009). Gaussian 09.

[44] Gavezzotti A (2007). OPiX: A Computer Program Package for the Calculation of Intermolecular Interactions and Crystal Energies.

[45] Khamitova DR, Blatov VA, Carlucci L, Ciani G, Proserpio DM (2009). Local and global topology of two-dimensional structural groups. Acta Crystallogr Sect A-Fundam Crystallogr.

