# Supplementary material for: The Hydrogen Bonded Structures of Two 5-Bromobarbituric Acids and Analysis of Unequal C5–X and C5–X′ Bond Lengths (X = X′ = F, Cl, Br or Me) in 5,5-Disubstituted Barbituric Acids
Source: Crystals (Basel). Author manuscript; Available in PMC 2017 Jun 30. (PMC5493196; doi:10.3390/cryst6040047)
Supplement: Supplementary material [file NIHMS73162-supplement-Supplementary_material.pdf]

crystals

Supplementary Materials: The Hydrogen Bonded Structures of Two 5-Bromobarbituric Acids and Analysis of Unequal C5–X and C5–X′ Bond Lengths (X = X′ = F, Cl, Br or Me) in 5,5-Disubstituted Barbituric Acids

Thomas Gelbrich [1,*], Doris E. Braun [1], Stefan Oberparleiter [2], Herwig Schottenberger [2] and Ulrich J. Griesser [1]

1. PXRD Characterization of the Desolvation Product of 1MH

1.1. Results

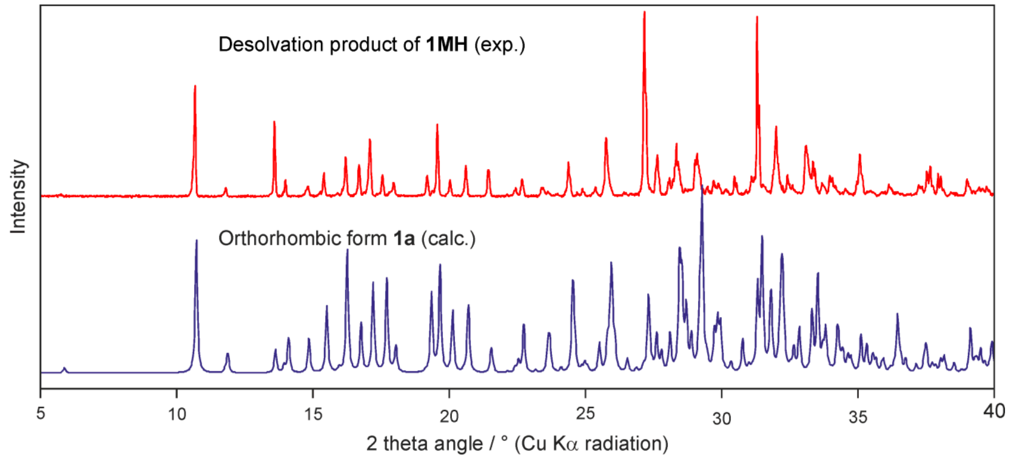

Figure S1. The experimental PXRD pattern of the desolvation product of **1MH** (top) in comparison with a simulated PXRD pattern based on the structure model of the orthorhombic polymorph **1a** [1] (bottom).

1.2. Experimental

The powder X-ray diffraction (PXRD) pattern of the desolvation product was obtained with an X′Pert PRO diffractometer (PANalytical, Almelo, NL) equipped with a theta/theta coupled goniometer, programmable XYZ stage with well plate holder (CuKα radiation source with focusing mirror; 0.5° divergence slit and 0.02° Soller slit collimator on the incident beam side; 2mm antiscattering slit and 0.02° Soller slit collimator on the diffracted beam side; solid state PIXcel detector). The pattern was recorded at a tube voltage of 40 kV and a tube current of 40 mA, using a 2θ scan rate of 0.013°/s in the range of 2° ≤ 2θ ≤ 40°.

2. H-Bonded Structure of 1MH

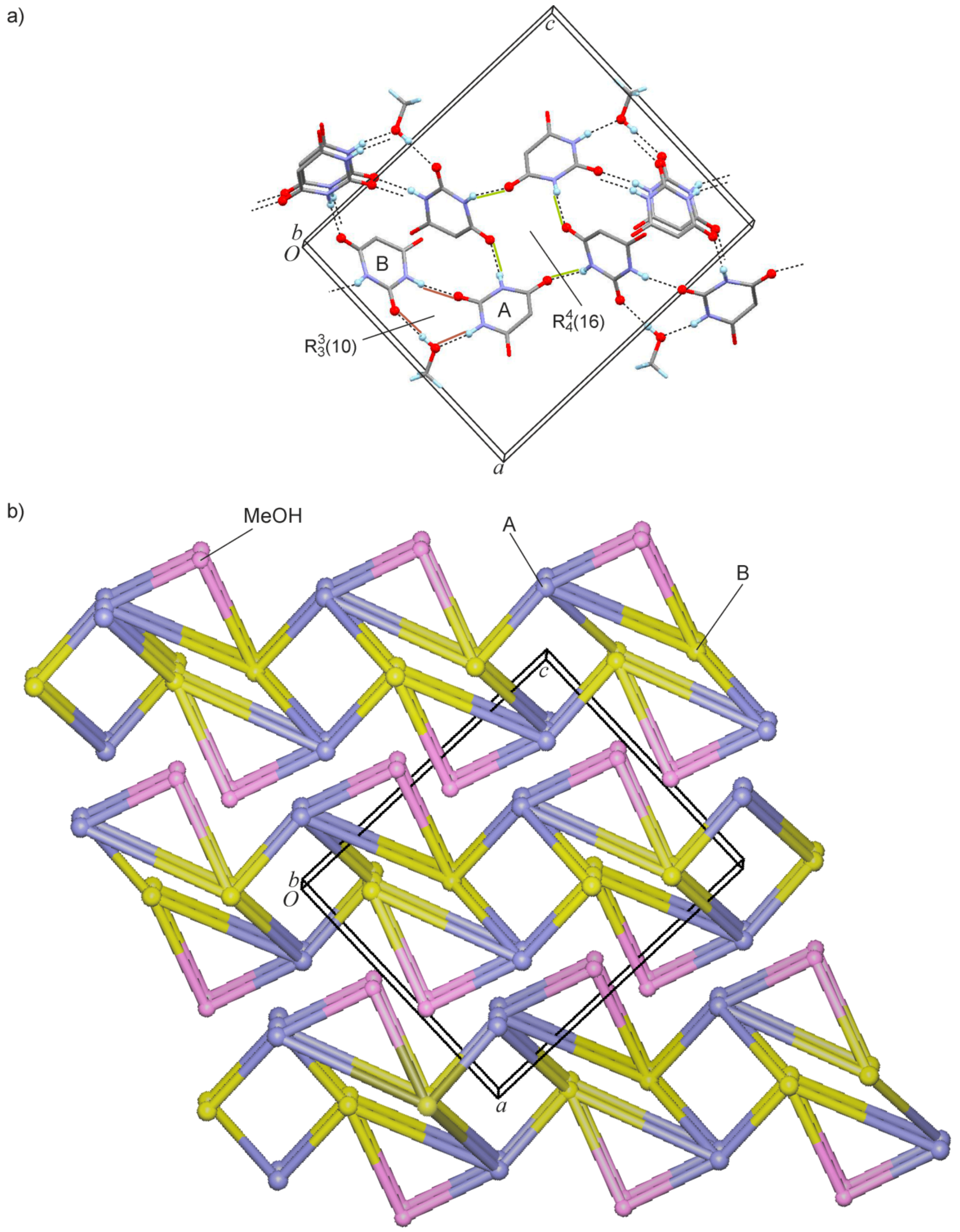

Figure S2. (**a**) N–H···O=C, N–H···O(MeOH) and (MeOH)O–H···O=C-bonded layer structure of **1MH**. O and H atoms involved in hydrogen bonding are drawn as spheres and hydrogen bonds are drawn as dashed lines. Br atoms are omitted for clarity. (**b**) Analogous view of the underlying topological nets of three instances of the H-bonded layer structure in the crystal of **1MH**.

3. SCDS-PIXEL Calculation for 2

The total PIXEL energy of the crystal, $E_{T,Cry}$, is –152.3 kJ·mol^{-1}.

Table S1. Total PIXEL interaction energies, E_T (kJ mol^{-1}), for the 14 most important pairwise interactions of **2**, partitioned into contributions from Coulombic (E_C), polarization (E_P), dispersion (E_D) and repulsion (E_R) terms. Symmetry equivalence of two interactions is indicated by a prime (*e.g.* #1/1′). *d* is the distance between the centroids of the two molecules involved. The right-hand column contains the percentage value of E_T relative to the sum of all PIXEL interaction energies of the crystal, $E_{T,\Sigma}$.[a]

#	Symmetry Operation	*d* (Å)	E_C	E_P	E_D	E_R	E_T	E_T / $E_{T,\Sigma}$
1	*x* – 0.5, –y + 0.5, 2 – *z*	6.45	–42.0	–19.4	–36.6	45.5	–52.5	16.6%
1'	*x* + 0.5, –y + 0.5, 2 – *z*	6.45	–42.0	–19.4	–36.6	45.5	–52.5	16.6%
3	*x* – 1, *y*, *z*	6.84	–31.3	–13.4	–29.2	43.4	–30.5	9.6%
3'	*x* + 1, *y*, *z*	6.84	–31.3	–13.4	–29.2	43.4	–30.5	9.6%
5	–*x* + 1.5, 1 – *y*, *z* – 0.5	10.11	–26.5	–10.0	–14.2	22.9	–27.8	8.8%
5'	–*x* + 1.5, 1 – *y*, *z* + 0.5	10.11	–26.5	–10.0	–14.2	22.9	–27.8	8.8%
7	–*x* + 0.5, 1 – *y*, *z* – 0.5	8.67	–7.3	–1.8	–19.1	7.6	–20.6	6.5%
7'	–*x* + 0.5, 1 – *y*, *z* + 0.5	8.67	–7.3	–1.8	–19.1	7.6	–20.6	6.5%
9	*x* – 0.5, 1.5 – y, 2 – *z*	6.18	–6.0	–3.2	–27.4	16.8	–19.9	6.3%
9'	*x* + 0.5, 1.5 – y, 2 – *z*	6.18	–6.0	–3.2	–27.4	16.8	–19.9	6.3%
11	–*x*, *y* – 0.5, 1.5 – *z*	11.10	–0.9	0.0	–0.7	0.0	–1.7	0.5%
11'	–*x*, *y* + 0.5, 1.5 – *z*	11.10	–0.9	0.0	–0.7	0.0	–1.7	0.5%
13	1 – *x*, *y* – 0.5, 1.5 – *z*	10.18	–16.9	–4.9	–11.4	31.8	–1.5	0.5%
13'	1 – *x*, *y* + 0.5, 1.5 – *z*	10.18	–16.9	–4.9	–11.4	31.8	–1.5	0.5%

[a] The polarization energy is not pairwise additive (many-body effect) so that the total PIXEL energy for the crystal, $E_{T,Cry}$, differs from the sum of all individual PIXEL interaction energies $E_{T,\Sigma}$ of –158.6 kJ mol^{-1}. #1/1′ = two-point interaction N1–H···O2(*x* – 0.5, –y + 0.5, 2 – *z*) and N3′–H···O6(*x* + 0.5, –y + 0.5, 2 – *z*); #3/3′ = one-point interaction N3–H···O6(*x*, *y* + 1, *z*); #5/5′ = one-point interaction N1′–H···O2(–*x* + 1.5, 1 – *y*, *z* + 0.5)

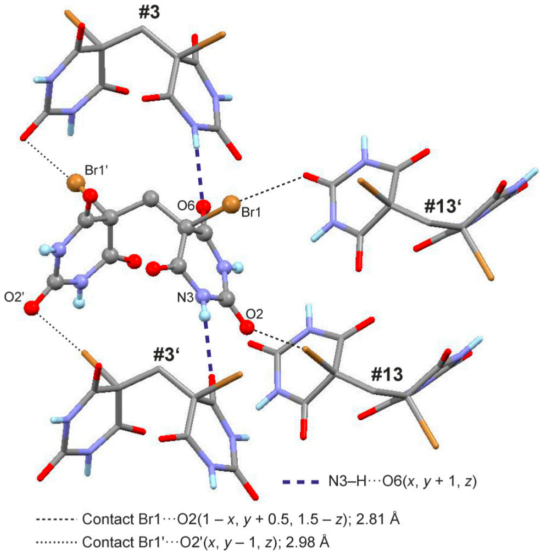

Figure S3. General molecule in the crystal structure of **2** (drawn in balls and sticks style) and four neighboring molecules (#3/3′ and #13/13′) in two symmetry-equivalent interaction pairs which result in two types of short Br···O distances. The interaction #3/3′ additionally involves the N3–H···O6(*x*, *y* + 1, *z*) bond (gray = C, light blue = H, blue = N, red = O, brown = Br).

4. Surveys of the Cambridge Structural Database

4.1. C–Br Distances in Br–C–Br Fragments

The Cambridge Structural Database (CSD; version 5.37 [2]) was searched for Br–C(sp^3)–Br fragments [excluding –CBr_3; no solvent molecules; *R* < 0.075; not disordered; no errors; not

polymeric; no ions; no powder structures; only organics]. This survey resulted in hits for 250 crystal structures containing 332 distinct Br–C–Br fragments with two C–Br bonds each (d_1 and d_2; $d_1 \geq d_2$). The corresponding data points (circles) are plotted in the diagram below, together with data points for the 5,5-dibromobarbituric acid (**1**) (filled triangles) phases **1MH**, **1a** and **1b** and for a molecular complex of **1** with melamine [3]. For 271 out of the 332 bond distance pairs, the difference (d_1–d_2) is 0.03 Å or lower. The plot indicates that the deviations found between the two types of C–Br bond distance in 5,5-dibromobarbituric acid are both systematic and unusually large. The average C–Br bond length for the whole set is 1.924 Å.

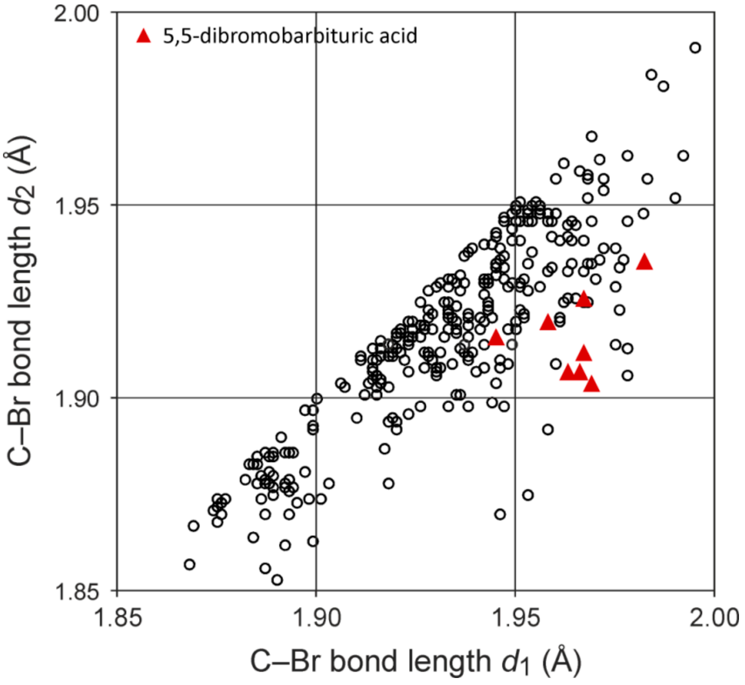

Figure S4. Bond distances d_2 plotted against the corresponding d_1 distances for 332 Br–C(sp^3)–Br structure fragments from the CSD and from this study. Red triangles represent the data points for solid forms containing the 5,5-dibromobarbituric acid molecule.

4.2. Correlation Between C–C–X Bond Angle and C–X Bond Length (X = Br, Cl, F, Me)

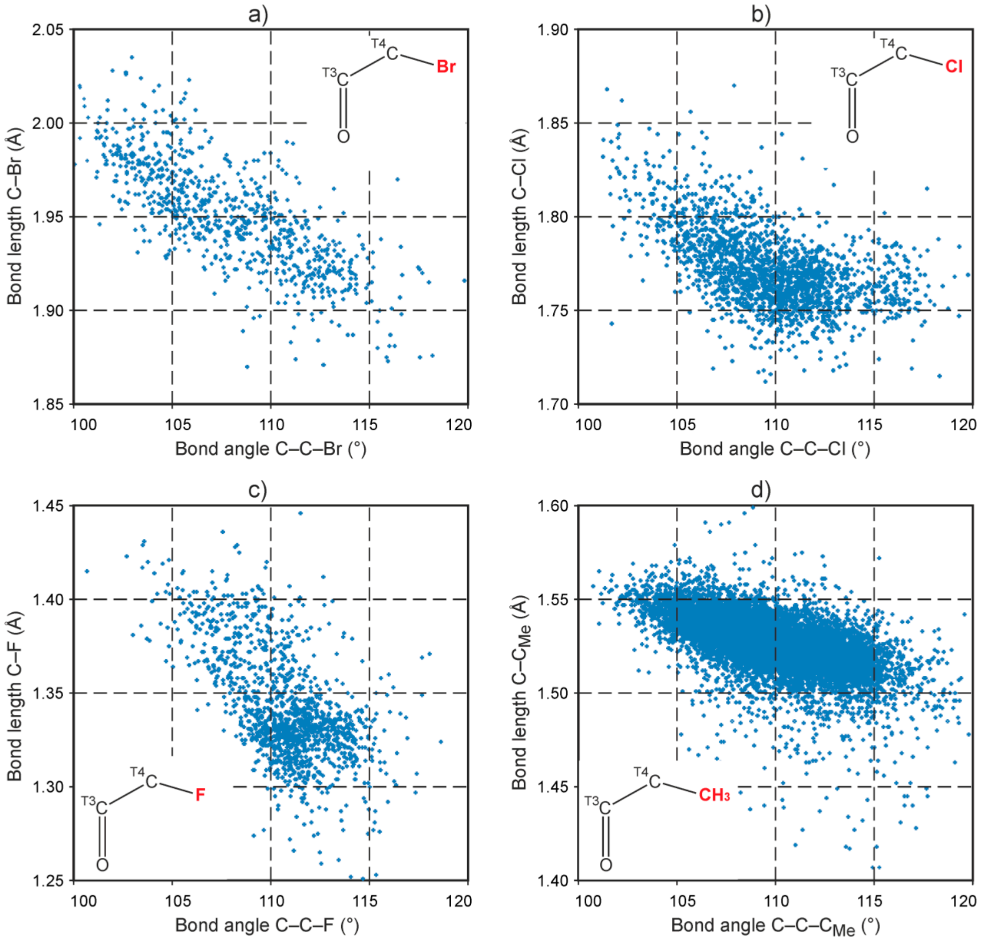

Figure S5. C–X bond lengths plotted against C–C–X bond angles for molecular fragments O=C(sp²)–C(sp³)–X (inset in upper right-hand corner) contained in the CSD (no disorder, no errors, not polymorphic, no ions, no powder structures, only organics) with a) X = Br (819 fragments from 541 crystal structures with *R* < 0.075), b) X = Cl (966 fragments from 693 crystal structures with *R* < 0.05), c) X = (1347 fragments from 501 crystal structures with *R* < 0.05) and d) X = Me (11823 fragments from 1343 crystal structures with *R* < 0.05).

5. Formation of Compound 2

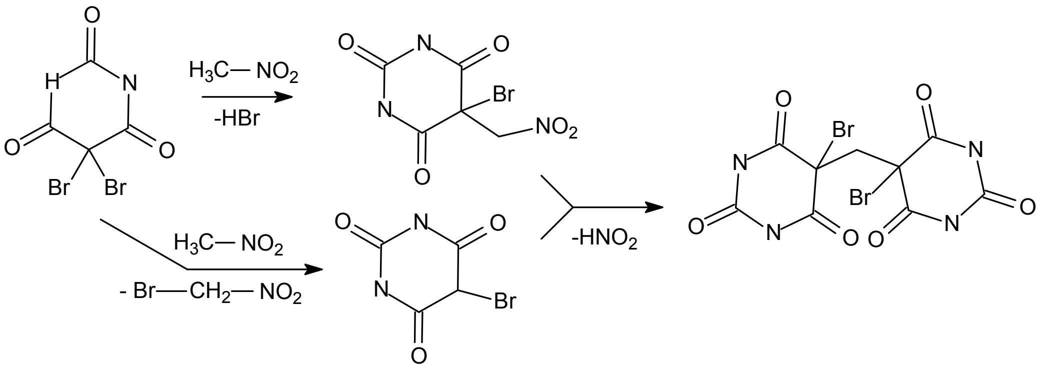

Scheme S1. Proposed trace formation of **2** in moist nitromethane.

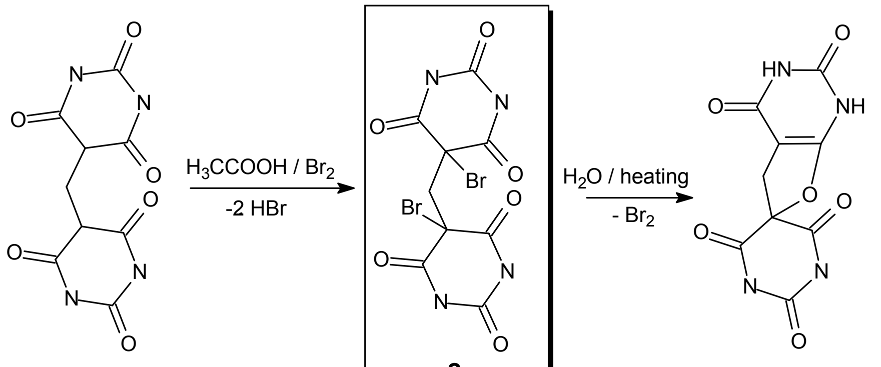

[27406-39-9] **2** [1333529-88-6]

Scheme S2. Targeted synthesis of **2** and subsequent conversion to a stable spiro enol ether [4,5].

Experimental

All chemicals were purchased from Sigma-Aldrich, St. Louis, MO, USA (European affiliate, Steinheim, Germany). The IR spectrum was obtained with a Nicolet 5700 FT spectrometer (Thermo Fisher Scientific Inc., Waltham, MA, USA) in ATR mode.

Preparative procedure of **2**. 5,5'-Methylenebis-2,4,6(1*H*,3*H*,5*H*)-pyrimidinetrione, [27406-39-9], (0.67 g, 0.0025 mol) was placed in a 250 mL round bottom flask and partially dissolved in CH_3COOH (15 mL) by magnetical stirring. Subsequently, still under stirring at room temperature, a solution of Br_2 (1.12 g, 0.0050 mol) in CH_3COOH (50 mL) was added through a dropping funnel over a period of 20 minutes. During the addition of the bromine in glacial acetic acid, any suspended material completely dissolved, resulting in a homogeneous yellow-orange reaction mixture. Stirring was continued for an another hour, and then all volatiles were stripped off by means of a rotary evaporator. Final evacuation of the crude remainder under oil-pump vacuum yielded a white microcrystalline powder of **2** (1.06 g; 100 % of theory).

Note: On exposure of **2** to adventive humidity, as well as in contact with moist or heated solvents, due to instantaneous liberation of bromine, a typical gradual color change from off-white to yellow to red-orange can be observed, which indicates the subsequent conversion of **2** to the spiro enol ether 1,5-dihydro-spiro[furo[2,3-*d*]pyrimidine-6(2*H*),5'(2'*H*)-pyrimidine]-2,2',4,4',6'(1'*H*,3*H*,3'*H*)-pentone [1333529-88-6]. M.p. 190 °C (decomposition). IR (neat): $\tilde{\nu}$ 3246 (br), 1687 (s), 1437 (w), 1348 (s), 1302 (vw), 1144 (m), 1120 (m), 1092 (w), 762 (s), 709 (s), 630 (m), 509 (s), 481 (s), 468 (s), 406 (s) cm^{-1}.

6. References

1. Gelbrich, T.; Rossi, D.; Häfele, C.A.; Griesser, U.J. Barbiturates with hydrogen-bonded layer and framework structures. *CrystEngComm* **2011**, *13*, 5502–5509.
2. Groom, C.R.; Allen, F.H. The Cambridge Structural Database in retrospect and prospect. *Angew. Chem. Int. Ed.* **2014**, *53*, 662–671.
3. Zerkowski, J.A.; MacDonald, J.C.; Whitesides, G.M. Investigations into the robustness of secondary and tertiary architecture of hydrogen-bonded crystalline tapes. *Chem. Mater.* **1994**, *6*, 1250–1257.
4. Jalilzadeh, M.; Pesyan, N.N.; Rezaee, F.; Rastgar, S.; Hosseini, Y.; Sahin, E. New one-pot synthesis of spiro[furo[2,3-d]pyrimidine-6,5'-pyrimidine]pentaones and their sulfur analogues. *Mol. Diversity* **2011**, *15*, 721–731.
5. Ara, T.; Khan, K.Z. Synthesis of some derivatives of dimedone, γ-pyrone and barbituric acid. *J. Pharm. Res. (Mohali, India)* **2014**, *8*, 786–790.

© 2016 by the authors; licensee MDPI, Basel, Switzerland. This article is an open access article distributed under the terms and conditions of the Creative Commons by Attribution (CC-BY) license (http://creativecommons.org/licenses/by/4.0/).
